# Gene‐Hydrogel Microenvironment Regulates Extracellular Matrix Metabolism Balance in Nucleus Pulposus

**DOI:** 10.1002/advs.201902099

**Published:** 2019-10-07

**Authors:** Wei Chen, Hao Chen, Dandan Zheng, Hongbo Zhang, Lianfu Deng, Wenguo Cui, Yuhui Zhang, Hélder A. Santos, Hongxing Shen

**Affiliations:** ^1^ Department of Spine Surgery Renji Hospital Shanghai JiaoTong University School of Medicine 160 Pujian Road Shanghai 200127 P. R. China; ^2^ Shanghai Key Laboratory for Prevention and Treatment of Bone and Joint Diseases Shanghai Institute of Traumatology and Orthopaedics Ruijin Hospital Shanghai Jiao Tong University School of Medicine 197 Ruijin 2nd Road Shanghai 200025 P. R. China; ^3^ Pharmaceutical Sciences Laboratory and Turku Bioscience Center Åbo Akademi University Turku FI‐20520 Finland; ^4^ Drug Research Program Division of Pharmaceutical Chemistry and Technology Faculty of Pharmacy University of Helsinki Helsinki FI‐00014 Finland; ^5^ Helsinki Institute of Life Science (HiLIFE) University of Helsinki Helsinki FI‐00014 Finland

**Keywords:** Agomir, extracellular matrix, gene therapy, gene‐hydrogel microenvironment, hydrogels

## Abstract

Gene therapy provides an ideal potential treatment for intervertebral disk degeneration by delivering synthetic microRNAs (miRNAs) to regulate the gene expression levels. However, it is very challenging to deliver miRNAs directly, which leads to inactivation, low transfection efficiency, and short half‐life. Here, Agomir is loaded in hydrogel to construct a gene‐hydrogel microenvironment for regulating the synthesis/catabolism balance of the tissue extracellular matrix (ECM) to treat degenerative diseases. Agomir is a cholesterol‐, methylation‐, and phosphorothioate‐modified miRNA, which can mimic the function of miRNA to regulate the expression of the target gene. Agomir874 that mimics miRNA874 is synthesized to down regulate the expression of matrix metalloproteinases (MMPs) in nucleus pulposus (NP). At the same time, a polyethylene glycol (PEG) hydrogel is synthesized through Ag‐S coordination of 4‐arm PEG‐SH and silver ion solution, which has injectable, self‐healing, antimicrobial, degradable, and superabsorbent properties and matches perfectly with the mechanism of intervertebral disk. By delivering Agomir‐loaded PEG‐hydrogel to a degenerative intervertebral disk, a gene‐hydrogel microenvironment is constructed in situ, which reduces the expression of MMPs, regulates the synthesis/catabolism balance of ECM in the NP of the intervertebral disk, and improves the tissue microenvironment regeneration.

## Graphical abstract

In degenerative diseases, microenvironment deterioration is often followed by a vicious circle of cell death and aggravation of microenvironment deterioration. To break the cycle, a gene‐hydrogel microenvironment is constructed in situ, which regulates the synthesis/catabolism balance of extracellular matrix in the nucleus pulposus of the intervertebral disk and improves the tissue microenvironment regeneration.

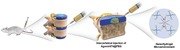

## 1 Introduction

In the past decade, it has been increasingly recognized that microRNAs (miRNAs) are an important part of gene regulatory networks by regulating the expression of target genes after transcription.^1^, ^2^, ^3^, ^4^ Gene therapy provides an ideal potential tool for many diseases by delivering synthetic miRNAs to regulate gene expression.^5^, ^6^, ^7^ Several vectors have been developed to deliver miRNAs to increase gene transfection, such as viral vectors, liposomes, and other vector systems. However, the biological toxicity, cellular immunoreactivity, and tumorigenicity of viral vectors limit their application in vivo.^8^, ^9^ Nonviral vectors have attracted more attention due to their easy synthesis, low immune response, high biocompatibility, and improved safety. Agomir is a cholesterol‐, methylation‐, and phosphorothioate‐modified miRNA fragment, which can mimic the function of miRNAs to regulate the expression of target genes.^10^ After cholesterol modification, the tail of miRNAs can form micelles to promote cellular uptake. Agomir has good stability and enhanced transfection efficiency in animals, and can penetrate the barriers of cell membrane and tissue in vivo to enrich target cells.^11^ Different Agomir structures based on different miRNA structures can effectively regulate the expression of target genes, thereby playing a role in the treatment of different diseases.

Intervertebral disk degeneration (IVDD) is a cascade reaction caused by the change of the nucleus pulposus (NP) microenvironment and imbalance of synthesis/catabolism of extracellular matrix (ECM).^12^, ^13^ The expression of matrix metalloproteinases (MMPs) increases during the process of disk degeneration, which facilitates the catabolism of ECM and leads to IVDD.^14^, ^15^ In the process of IVDD, abnormal expression of different miRNAs is often detected.^16^ MiRNAs can regulate the expression of MMPs in NP, which may contribute to the development of IVDD.^17^ While, few studies have been done on treating IVDD with miRNAs and Agomir has never been used. Herein, we are trying to find an miRNA to regulate MMPs gene expressions, and then synthesize the Agomir based on the miRNA structure to achieve a persistent and effective reduction of MMPs activity and ECM degradation.

Because of the avascular structure of intervertebral disk,^18^ systemic administration of miRNA is not effective for IVDD. Alternatively, intervertebral injection may better maintain the miRNA at the site of action, to achieve a better effect of slowing or inhibiting the progress of IVDD. However, the intervertebral disk connects two adjacent vertebral bodies and is surrounded by ligaments and muscles, which makes the intervertebral disk an important load‐bearing structure of vertebrates subjecting to forces in different directions at any time. The liquid therapeutics is easy to leak out of the intervertebral space from the pinhole, which reduces the curative effect of drugs and causes leakage‐related complications.^19^, ^20^ Therefore, biomaterials are used to deliver and maintain drugs for local treatment of intervertebral. Currently, there are mainly two types of biomaterials for intervertebral disk repair: biomaterials with high mechanical strength and biomaterials with certain elasticity and softness. The former ones include plastic and metal materials, which are mostly used in the surgical treatment of IVDD.^21^, ^22^ However, those materials have too high mechanical strength that may induce cartilage endplate damage and secondary degeneration to adjacent intervertebral disk segments. The latter ones include hydrogels and other materials, which are not compressive and have poor storage and dissipation properties.^23^, ^24^ Under pressure, they have the tendency to break into small fragments and damage the intervertebral disk, and have the risk of inducing the fibrillation of the intervertebral disk.^25^, ^26^ Therefore, a novel injectable hydrogel is necessary to be developed for delivering Agomir into the intervertebral space to slow or inhibit the progress of IVDD.

**Scheme 1:**
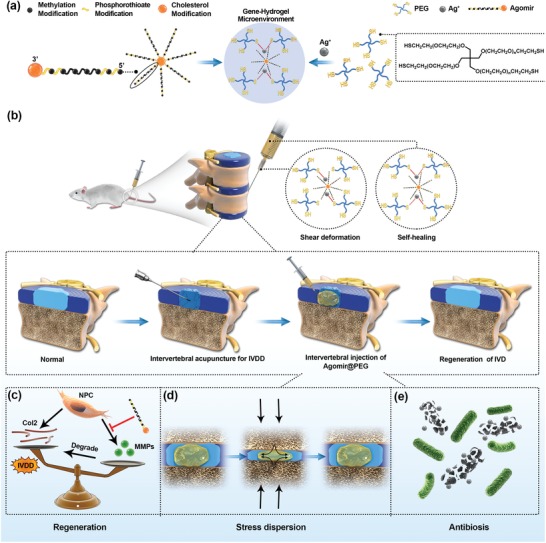
Gene‐hydrogel microenvironment for regeneration of IVDD. a) The construction of gene‐hydrogel microenvironment. b) The Agomir@PEG was injected into the intervertebral space to construct the gene‐hydrogel microenvironment. c–e) The multi‐functions provided by the gene‐hydrogel microenvironment, matching the regeneration of IVDD.

In this work, a multifunctional polyethylene glycol (PEG)‐hydrogel is synthesized by cross‐linking 4‐arm SH‐PEG with Ag^+^ via Ag–S coordination to have injectable and self‐healing properties.^27^, ^28^ The PEG‐hydrogel has favorable Agomir loading capacity and can be injected into intervertebral space by minimal invasive method. Moreover, the hydrogel can self‐heal to construct gene‐hydrogel microenvironment in situ after injection, which is supposed to make the hydrogel resistant to leakage and broken. And the mechanical strength of the PEG‐hydrogel is adjustable to match the condition of intervertebral disk. By delivering an Agomir, synthesized by special chemical modification based on the structure of miRNA whose expression is reduced in IVDD, the gene‐hydrogel microenvironment is constructed in situ by intervertebral space injection of Agomir loaded in PEG‐hydrogel. And the radiology and histology images harvested from animals are used to assess the effect of the gene‐hydrogel microenvironment (**Scheme** 1).

## 2 Results and Discussion

MiRNA is an important component of the gene regulatory network, which can regulate the expression of target gene after transcription and participate in the occurrence of many diseases. Transmitting synthetic miRNAs to regulate the gene expression provides an ideal potential therapy for many diseases. However, in consideration of the transfection efficiency and cytotoxicity, there are limitations in the use of existing carriers. Agomir is an ideal miRNA agonist for direct chemical modification of miRNAs.^29^ Cholesterol modification makes it more affine to cells, methylation and thio‐modification provide a longer half‐life and less inactivation. Local injection of Agomir into living tissues and targeted gene therapy of target cells can achieve excellent transfection efficiency and lasting action cycle. However, there are risks of leakage and easy diffusion in some deep tissues, which decreases the effectiveness of the targeted therapy. Therefore, an injectable vector is needed to provide a good environment for RNA therapy. Injectable biomaterials are temperature‐ and pH‐sensitive. However, these materials have a physical or chemical change process on the material itself, some of which are even irreversible.

**Figure 1:**
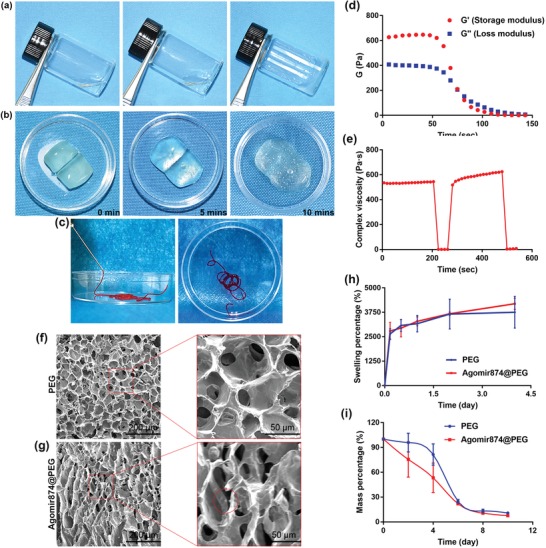
Properties of the PEG‐Ag hydrogels. a) The mixture of PEG‐thiol and AgNO_3_ solution. b) The process of self‐healing. c) Photograph of the injectable hydrogel. d) Strain sweep measurements of the storage moduli (*G*' denotes the elastic modulus and *G*” denotes the loss modulus). e) Measure of viscosity parameters in relation to time in seconds. The strain shearing rate alternated between 0.05% strain for 100 s and 500% strain for 50 s. f,g) Magnified SEM results (Agomir874 was marked by red circle). h) Swelling percentage. i) Degradation percentage.

In this study, we used a Ag^+^ and 4‐arm SH‐PEG to form a Ag–SH coordination bond. After mixing the two solutions, they quickly gelled to form a translucent, water‐rich jelly‐like hydrogel with a soft and sticky texture (**Figure** 1a). This Ag–SH coordination bond does not require any catalyst and can react spontaneously, which makes the hydrogel self‐healing.^30^, ^31^ When the whole hydrogel is cut into half, it can gradually merge into a whole hydrogel (Figure 1b) in 10 min. At the same time, because the Ag–SH coordination will break under the action of larger shear force, PEG‐Ag hydrogel can smoothly pass through a 1 mL syringe (Figure 1c) to complete the injection of deep tissue. Rheological analysis of hydrogels revealed that the storage modulus *G*′ of PEG‐Ag hydrogel under a static condition was about 600 Pa, and the loss modulus *G*″ was about 400 Pa. When the hydrogel was applied with a large shear force, the storage modulus and loss modulus decreased to 0 Pa (Figure 1d). At the same time, the composite viscosity of the hydrogel was about 550 Pa s when no force was applied, indicating that the state of the hydrogel was still in a solid state at this time. When a larger shear force was applied, the composite viscosity decreased to 0 Pa s, indicating that the hydrogel is in a fluid state at this time. Once the force is removed, the composite viscosity of the hydrogel rose again to about 600 Pa s, indicating that the hydrogel becomes solid again at this time (Figure 1e). The PEG‐Ag hydrogel is gelled by spontaneous formation of coordination bond between Ag–SH. Under the action of shear force, the bond will break reversibly, resulting in the transformation of the hydrogel into a fluid state, which makes the hydrogel injectable. At the same time, the hydrogel can spontaneously coordinate to form Ag–SH bond again after injection, and thus, the hydrogel can self‐heal in situ and provide a good environment for genes and drugs.

Hydrogels loaded with water‐soluble drugs need to have a strong hygroscopic property, which can absorb a large amount of water and load drugs at the same time. The PEG‐Ag hydrogel has a porous and multi‐void structure, as shown in the scanning electron microscopy (SEM) images (Figure 1f). Each void communicates with each other. The structure provides the hydrogel a good hygroscopic property. After incubation in phosphate‐buffered saline (PBS), the swelling mass of the hydrogel can reach more than 3000% and keeps its water content up to a week. After Agomir874 was added, the microstructure of the hydrogel remains unchanged and present a loose multi‐void structure (Figure 1g) with an improved hygroscopic property (Figure 1h). The structure of PEG‐Ag hydrogel makes it biodegradable. In PBS aqueous solution, PEG‐Ag can maintain a certain quality in 4 days, while the degradation rate of PEG‐Ag hydrogel accelerates from the fourth day, and only 20% of the mass remains undegraded after 8 days (Figure 1i). This degradation rate can provide a stable microenvironment for Agomir in vivo within the first few days, and when Agomir plays a regulatory role, it degrades, reduces its influence on the ECM synthesis, and promotes the tissue regeneration.

**Figure 2:**
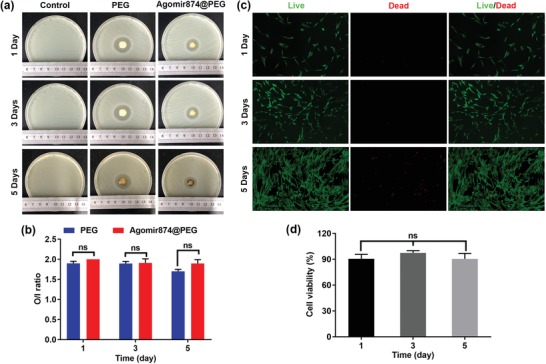
Biological study of hydrogels in vitro. a,b) Antibacterial sensitivity with agar diffusion test. c,d) Live/dead staining of human fibroblasts co‐cultured with PEG‐Ag hydrogel.

The process of injecting drug is a minimally invasive operation, however there still exists a certain risk of infection. Therefore, special attention should be paid to aseptic techniques.^32^ Fortunately, PEG‐Ag hydrogels are rich in Ag ions, which can destroy sulfur‐containing proteins in the bacteria's cell membranes and kill them.^33^, ^34^ The sensitivity of *Staphylococcus aureus* ATCC25923 to Agomir874‐loaded PEG hydrogels and PEG‐hydrogels was determined by a method similar to Kirby–Bauer disk diffusion test. The antimicrobial activity (**Figure** 2a) of PEG‐hydrogels with or without Agomir874 was observed, and the addition of Agomir 874 did not affect the antimicrobial activity of PEG‐hydrogels (Figure 2b).

The biocompatibility is the primary prerequisite for the application of biomaterials.^35^ Primary NP cells (NPCs) were extracted from coccygeal intervertebral disk of rats (Figure S1, Supporting Information). Living/dead cell staining showed that PEG‐Ag hydrogel had little toxicity to human fibroblasts (Figure 2c), and the cell activity was close to 100% in 1, 3, and 5 days, with no statistically significant differences (Figure 2d). At the same time, the exudate of 1, 2, 4 days hydrogel was used to carry out Cell Counting Kit‐8 test on rat NPCs and mouse osteoblast precursor 3T3 cells. It was found that the exudates of different days had no obvious biological toxicity to rat NPCs and mouse osteoblast precursors (Figure S2, Supporting Information). This suggests that the hydrogel can be well compatible in different species and tissues without cytotoxicity and can be widely used in various tissue regeneration processes.

**Figure 3:**
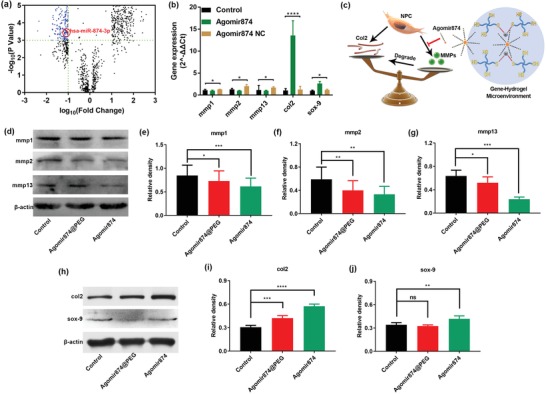
The effects of Agomir874@PEG on regulating the synthesis/catabolism balance of NPCs in vitro. a) The volcano plot of differential expression of miRNAs in IVDD. Red circles denote miRNA874 under‐expressed in degenerative intervertebral disc. b) Polymerase chain reaction data of gene expression in NPCs treated with Agomir874 or Agomir874 NC. c) The diagram of the effects of gene‐hydrogel microenvironment on regulating the synthesis/catabolism balance. d) Western blotting data showing the levels of down‐regulating the expression of MMPs. Western blotting data of the levels of e) MMP‐1 and f) MMP‐2 and g) MMP‐13. h) Western blotting data showing the levels of up‐regulation of the expression of Col II and Sox‐9. Western blotting data of the levels of i) Col II and (j) Sox‐9.

The key pathological mechanism of degenerative diseases in human body is that the balance of synthesis/catabolism of the ECM is broken. Oversynthesis or degradation of different ECM leads to abnormal proliferation or damage of various tissues, which results in various diseases.^36^ Overexpression of MMPs during degeneration leads to excessive degradation of the ECM of NP, especially type II collagen, resulting in disk degeneration, such as loss of water, loss of intervertebral space height, and rupture of the NP. Differential expression of miRNAs in degenerative NP tissues was investigated by the data in National Center for Biotechnology Information Gene Expression Omnibus database (GSE116726).^37^ Of 2570 miRNAs detected, miRNA874 was under expressed in the degenerative NP tissues compared with the controls (**Figure** 3a). Activating the expression of miRNA874 in NPCs by treating with Agomir874 can down‐regulate the expression of MMPs and increase the expression of ECM components, especially type II collagen (Figure 3b). Therefore, in the early stage of IVDD, Agomir874 based on the structure of miRNA874 is able to achieve a persistent and effective reduction of MMPs activity and ECM degradation (Figure 3c). Under the action of Agomir874, the expression of MMPs in NPCs was down‐regulated in varying degrees (Figure 3d–g), especially in MMP‐2 and MMP‐13 (Figure 3f,g). MMP‐2 is a gelatinase‐like MMP. Its main function is to degrade denatured collagen and gelatin. MMP‐13 is an interstitial collagenase, which can degrade many types of collagen and most proteoglycan molecules. Agomir874 can negatively regulate the expression of MMPs, thereby reducing the degradation of ECM synthesized by NPCs and increasing the content of ECM (Figure 3h–j), especially the content of type II collagen (Figure 3i). Agomir874 can reverse the imbalance of synthesis/decomposition by regulating the balance of synthesis/decomposition of the ECM of NP and inhibit the degeneration of intervertebral disk. At the same time, the in vitro evaluation of NPCs treated by PEG‐Ag hydrogel‐loaded Agomir874 was similar to that by Agomir874 alone (Figure 3).

**Figure 4:**
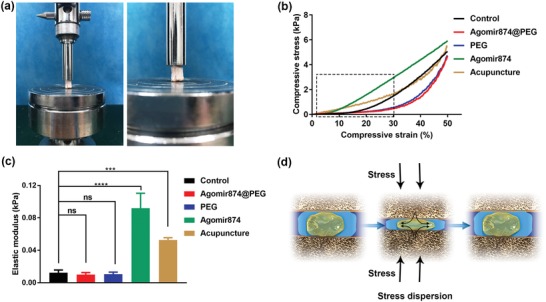
Single‐segment intervertebral biomechanical studies. a) The diagram of single‐segment intervertebral biomechanical studies. b) Compression curve of different treatment at single‐segment of intervertebral disk. c) The elastic modulus of the first 30% of the deformity rate. d) The schematic diagram of stress dispersion of gene‐hydrogel microenvironment.

Intervertebral disks can provide buffering force and flexibility to spine movement.^38^ Therefore, biomaterials injected into intervertebral disk should be in good harmony with mechanical properties of intervertebral disk, as well as providing buffering force and flexibility to spine movement. The results of single‐segment intervertebral biomechanical studies (**Figure** 4a) demonstrate that compared with the Agomir874 and acupuncture groups, the control and injected groups with hydrogel showed improved mechanical properties (Figure 4b). According to the range of normal physiological activities, we take the first 30% of the deformity rate and calculate the corresponding elastic modulus. The results show that there were no statistically significant differences between the groups injected with hydrogel and the control group. Moreover, there was a statistically significant increase of the elastic modulus in the acupuncture and the Agomir874 groups. This suggests that the mechanical properties of PEG‐hydrogel are similar to intervertebral disk. Single Agomir874, which exists in the form of liquid, tends to leak through the pinhole. This explains why the elastic modulus of the Agomir874 group is similar to the acupuncture group (Figure 4c). Also, the addition of PEG‐hydrogel provides extra buffer to adjacent segments and protects adjacent intervertebral disks from degeneration (Figure 4d).

**Figure 5:**
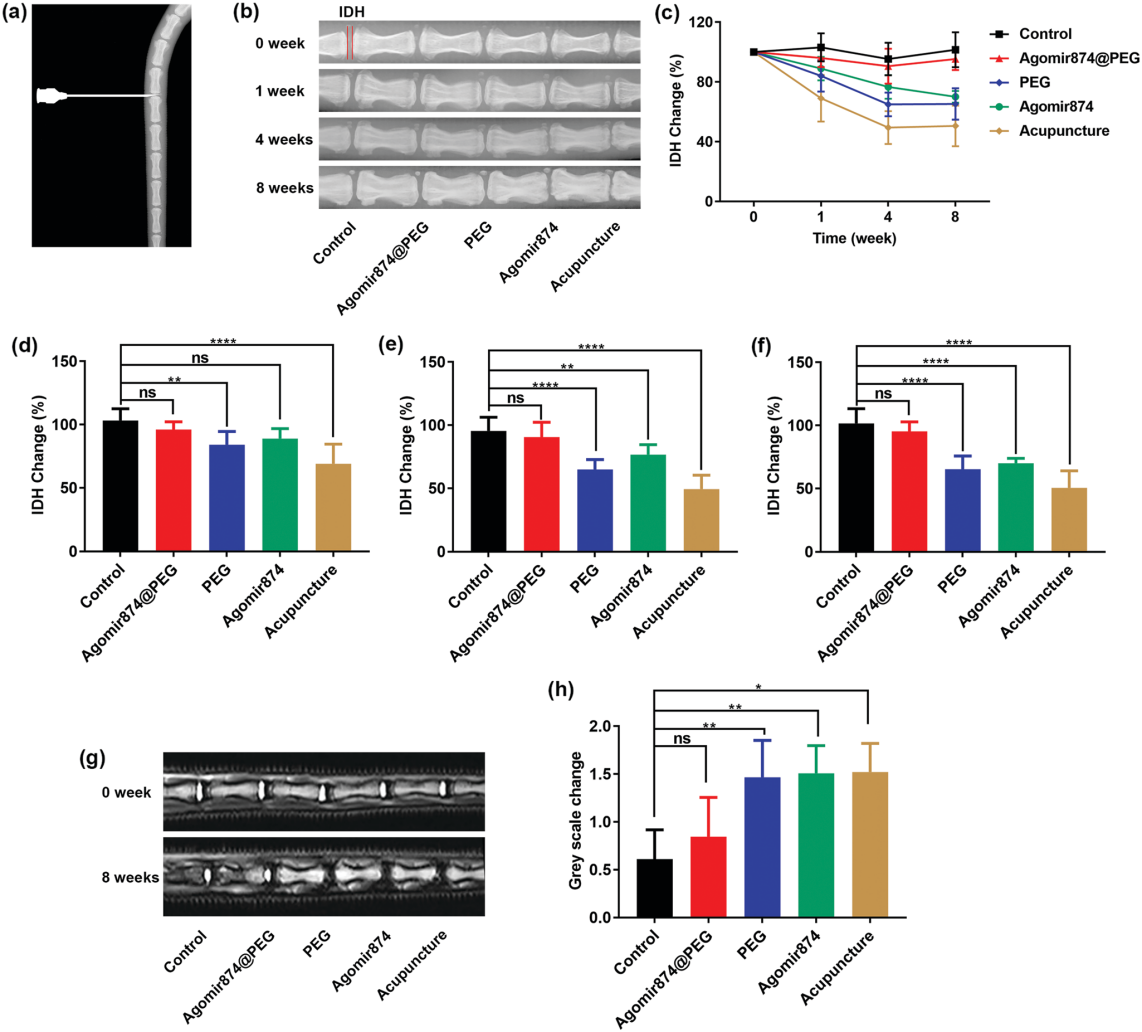
Imaging data of animal experiments. a) Acupuncture model of rat coccygeal vertebrae. b) The X‐ray images of rat coccygeal vertebrae. c) The intervertebral disc height (IDH) changes of different groups at different time points. d) The IDH changes of different groups at 1 week after surgery. e) The IDH changes of different groups at 4 weeks after surgery. f) The IDH changes of different groups at 8 weeks after surgery. g) The MRI images of rat coccygeal vertebrae. h) The gray scale changes of different groups at 8 weeks after surgery.

To further illustrate the in vivo effect of Agomir874‐loaded PEG‐Ag hydrogel, a rat model of IVDD was established as described elsewhere^39^, ^40^ (**Figure** 5a). Histologic sections and radiology evaluation were recorded at different time points. Disk heights can reflect the changes of ECM^41^ (Figure 5b). In the Agomir874@PEG group, rat IVDD gradually restores to normal height, which was similar to the control group. In the acupuncture group, rat IVDD gradually collapses. The results of the PEG‐Ag hydrogel and Agomir874 groups were similar to the acupuncture group, indicating that single PEG‐Ag hydrogel or Agomir874 solution had little therapeutic effects (Figure 5c). The results of different time points showed that the Agomir874@PEG group had no statistically significant difference compared with the control group, while the other group showed statistically significant lower disk heights (Figure 5d–f). Magnetic resonance imaging (MRI) can reflect the water content of IVDD,^42^, ^43^ and the higher gray scale indicates lower water content (Figure 5g). MRI scan performed 8 weeks after operation showed lower water content in the Agomir874@PEG group compared with the control group, however there were no statistically significant differences. The PEG‐Ag hydrogel and Agomir874 groups had significantly lower water content, similar to the acupuncture group.

**Figure 6:**
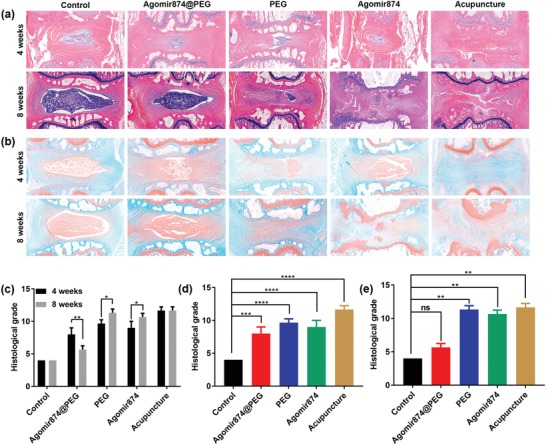
Histological images of animal experiments. a) The H&E staining images of different groups at different time points. b) The Safranin O staining images of different groups at different time points. c) The histological grade of different groups at different time points. d) The histological grade of different groups at 4 weeks. e) The histological grade of different groups at 8 weeks.

Histological sections were obtained 4 and 8 weeks after operation. Hematoxylin and eosin (H&E) staining was used to observe the NP, annulus fibrosus, and their margin. 8 weeks after operation, the Agomir874@PEG group showed clear border between the NP and annulus fibrosus, as well as significant improvement in the NP compared with 4 weeks. Furthermore, in the PEG‐Ag hydrogel, Agomir874, and acupuncture groups, a very vaguely defined border between the NP and annulus fibrosus was noted, along with worsened NP status (**Figure** 6a). Safranin O staining was used to observe collagen level in the NP. Compared with the control group, collagen level was significantly higher in the Agomir874@PEG group and lower in the PEG‐Ag hydrogel, Agomir874, and acupuncture groups (Figure 6b). The results of type II collagen immunohistochemistry demonstrated a normal expression of type II collagen and the intact NP in the Agomir874@PEG group. In the PEG‐Ag hydrogel and Agomir874 groups, however, the expression of type II collagen was nearly absent (Figure S3, Supporting Information). Histological score was calculated according to previous reports.^44^, ^45^ In the Agomir874@PEG group, the histological score was significantly lower 8 weeks after operation, indicating an inhibition of disk degeneration along with a process of regeneration. In the PEG‐Ag hydrogel and Agomir874 groups, the histological score increased over time. Also, at 4 weeks after operation, both the Agomir874@PEG, PEG‐Ag hydrogel and Agomir874, groups had higher histological score than the control group (Figure 6c). However, at 8 weeks post‐operation, there were no statistically significant differences between the Agomir874@PEG and control groups, indicating that this healing process occurs after disk degeneration and has reversal effect.

Although the PEG‐Ag hydrogel can provide structural support for intervertebral space at early stages, its excellent biodegradability leads to decomposition within the first week after implantation, and thus, cannot provide long‐term support. Single Agomir874 can inhibit the MMPs secretion in NPCs and regulate the metabolic balance, but abundant RNase existing in the intervertebral disk can be destructive to the Agomir874 solution. Also, liquid form Agomir874 tends to leak through the pinhole due to high pressure inside the intervertebral disk, leading to decreased amount of effective Agomir874. By loading Agomir874 into the PEG‐Ag hydrogel, we created a gene‐hydrogel microenvironment inside the intervertebral disk, which can effectively regulate the anabolism balance during IVDD and promote disk regeneration.

## 3 Conclusion

In conclusion, we demonstrated that Agomir874 based on the structure of miRNA874 can down‐regulate the expression of MMPs, thereby regulating the metabolism balance of ECM in NP and slowing the process of IVDD for the first time. At the same time, a multifunctional PEG‐hydrogel with injectable, self‐healing, antimicrobial, degradable, and superabsorbent properties was used as a novelty biomaterial to deliver the Agomir874 in IVDD. The mechanical performance of the PEG‐hydrogel in single‐segment intervertebral disk was discussed for the first time. It was proved that the PEG‐hydrogel has similar mechanical properties to normal intervertebral disk and can match and compensate for the elasticity of degenerated intervertebral disk. By injecting Agomir loaded in PEG‐hydrogel, a gene‐hydrogel microenvironment was constructed in situ, which was proved to regulate the balance of metabolism of ECM in NPC of intervertebral disk in vivo, and provide opportunities for intervertebral disk regeneration so as to achieve the effect of treating IVDD. We also believe that this gene‐hydrogel microenvironment can be applied in other diseases of which miRNAs contribute to the progress.

## Conflict of Interest

The authors declare no conflict of interest.

## Supporting information

Supplementary
