# Supplementary material for: Gene‐Hydrogel Microenvironment Regulates Extracellular Matrix Metabolism Balance in Nucleus Pulposus
Source: Adv Sci (Weinh). 2019 Oct 7;7(1):1902099. doi: 10.1002/advs.201902099 (PMC6947697; doi:10.1002/advs.201902099)
Supplement: Supplementary file 1 — Supplementary [file ADVS-7-1902099-s001.pdf]

Copyright WILEY-VCH Verlag GmbH & Co. KGaA, 69469 Weinheim, Germany, 2019.

Supporting Information

for *Adv. Sci.*, DOI: 10.1002/advs.201902099

Gene-Hydrogel Microenvironment Regulates Extracellular Matrix Metabolism Balance in Nucleus Pulposus

Wei Chen, Hao Chen, Dandan Zheng, Hongbo Zhang, Lianfu Deng, Wenguo Cui, Yuhui Zhang, Hélder A. Santos,* and Hongxing Shen**

Support Information

Gene-hydrogel microenvironment regulates extracellular matrix metabolism balance in nucleus pulposus

Wei Chen[1], Hao Chen[1], Dandan Zheng[1], Hongbo Zhang, Lianfu Deng, Wenguo Cui,

Yuhui Zhang[*], Hélder A. Santos[*], Hongxing Shen[*]

[*] Dr. W. Chen, Dr. H. Chen, Dr. D. Zheng, Dr. Y. Zhang, Dr. H. Shen
Department of Spine Surgery, Shanghai Renji Hospital, Shanghai JiaoTong University School of Medicine, 1630 Dongfang Road, Shanghai 200011, P. R. China
E-mail: shenhxgk@126.com; zhangyuhui2003@126.com

[*] Prof. H. Zhang, L. Deng, W. Cui
Shanghai Key Laboratory for Prevention and Treatment of Bone and Joint Diseases, Shanghai Institute of Traumatology and Orthopaedics, Ruijin Hospital, Shanghai Jiao Tong University School of Medicine, 197 Ruijin 2nd Road, Shanghai 200025, P. R. China

Prof. H. Zhang
Department of Pharmaceutical Sciences Laboratory,
Åbo Akademi University, FI-00520, Finland.

Prof. H. A. Santos
Drug Research Program, Division of Pharmaceutical Chemistry and Technology, Faculty of Pharmacy, University of Helsinki, Helsinki, FI -00014, Finland
and
Helsinki Institute of Life Science (HiLIFE), University of Helsinki, Helsinki, FI -00014, Finland
E-mail: helder.santos@helsinki.fi

Keywords: Gene Therapy, Agomir, Hydrogel, Gene-Hydrogel Microenvironment, Extracellular Matrix.

Methods and materials

1. Preparation and evaluation of PEG-Ag hydrogel

1.1. Preparation of PEG-Ag hydrogel

The PEG-Ag hydrogel was prepared as following: A solution of 4arm-PEG-SH was prepared by dissolving 100 mg of 4arm-PEG-SH in 500 μl of deionized water. 200 μl of $AgNO_3$ solution was diluted in 500 μl of deionized water. PEG-Ag hydrogel was formed seconds after mixing 4arm-PEG-SH solution with 4arm-PEG-SH. To prepare Agomir-874 loaded hydrogel, 4 nmol of Agomir-874 was dissolved in 500 μl of 4arm-PEG-SH solution, then by mixing 4arm-PEG-SH solution with 4arm-PEG-SH, the PEG-Ag hydrogel loaded with Agomir-874 was formed.

1.2. Evaluation of the injectability and self-healing nature of PEG-Ag hydrogel

The injectability and self-healing nature of PEG-Ag hydrogel were evaluated using a digital camera. To study the self-healing nature of PEG-Ag hydrogel, the hydrogel was sliced into two pieces and separated for 2 mm, photos were captured at 0 min, 5 min, and 10 min. The injectability was studied by injecting the ink-stained hydrogel using a 1 ml Syringe with 5 mm needle.

1.3. Morphology investigation by scanning electron microscopy (SEM)

The hydrogel was frozen at −80 °C and freeze-dried. The sample was gold-sputter coated before investigated under SEM (FEI USA).

1.4. Hygroscopicity of hydrogel

To study the hygroscopicity of the hydrogel, 1 ml of hydrogel was separated into 3 parts and freeze-dried. Dry weight (W_0) was recorded before adding 10 ml of PBS solution into each sample. The moist hydrogel was incubated in 37 °C and weighed at 10 min, 30 min, 1 h, 2 h, 4 h, and 8 h (W_t). Swelling ratio was calculated using the equation [$(W_t – W_0)/W_0$].

1.5. Biodegradability of the hydrogel

To study the biodegradability of the hydrogel, 1 ml of hydrogel was separated into 3 parts and weighed. Then 10 ml of PBS was added to each part of the hydrogel and incubated at 37 °C under slow stirring. The remaining hydrogel was weighed every 2 days (W_t). The biodegradation level was determined by the equation W_t/W_0.

1.6. Rheological analysis

The hydrogel was placed in a parallel plate (40 mm) before analyzing using a strain rheometer. The stress-strain test was established by applying a low strain (γ = 0.05%) to the hydrogel for the first 100 s, then a high strain (γ = 500%) for the next 50 s. Once the high strain was discontinued, a low magnitude strain (γ = 0.05%) was applied.

2. In vitro experiments

2.1. Extraction and culture of nucleus pulposus cell (NPC)

The nucleus pulposus cells (NPC) used in this study were extracted from Sprague-Dawley rats (SD rats). Caudal vertebrae was separated from SD rats under sterile conditions. Nucleus pulposus was extracted and incubated in 0.1% Type II collagenase under 37 °C for 1 h. After incubation, 70 μm filter was used to remove the tissue debris. After centrifuge, the collagenase was removed, and NPC cells were cultured in Dulbecco's modified Eagle medium (DMEM) with 10% fetal bovine serum (Gibco) and 100 U/ml penicillin–streptomycin. Finally, NPCs were incubated at 37 °C in a humid atmosphere with 5% CO_2. The medium was changed every 72 h and low passage NPCs (P2−P4) were used in in vitro studies.

2.2. Antibiotic test

The susceptibility of *Staphylococcus aureus* ATCC25923 to PEG-Ag hydrogel and PEG-Ag hydrogel loaded with Agomir874 was determined by methods similar to Kirby-Bauer disk diffusion test (Oxoid, United Kingdom). Briefly, 50 mg of PEG-SH with or without Agomir874 was dissolved in 1 ml of deionized water and $AgNO_3$ was added to form hydrogel. *Staphylococcus aureus* ATCC25923 was planted on Mueller-Hinton agar plate (MH plate) and hydrogel with or without Agomir874 was planted on the center of MH plate. MH plate planted with *Staphylococcus aureus* was used as blank control.

2.3. Live-dead assay

A Live/Dead kit (Life Tech, USA) was used in this study. The PEG-Ag hydrogels were cocultured with NPCs for 24 h before incubated with 500 μl of dye at 37 °C for 10 min. The cells were washed gently with PBS. Then the cells were incubated with working solution for 30–45 min under room temperature. After incubation, the working solution were removed and 10 μl PBS were added before observing under fluorescence microscope.

2.4. CCK-8 test

1 ml of the hydrogel was separated into 3 parts and immersed in DMEM for 1, 2 or 4 days. The DMEM was then collected. 100 μl of cell suspension was added to each well of the 96-well plate and incubated for 24 h at 37°C. Then 10 μl of the collected DMEM was added to each well and incubated for 24–72 h at 37°C. Finally, 10 μl of CCK8 solution was added to each well, incubated for 1 h at 37°C and analyzed by an ELISA kit.

2.5. Quantitative real-time PCR analysis

NPCs were laid in 6-well tissue culture plates at 10 ^ 5/ml density. After 24 hours, NPC was transfected with Agomir 874 or Agomir 874 NC according to manufacturer's instructions. After 48 hours of incubation, total RNA was extracted from cells by Trizol reagent (Invitrogen, US) according to the manufacturer's instructions. The cDNA was synthesized using the primescript RT kit (Takara, Japan)

according to the manufacturer's plan. After the reverse transcription reaction, rt-PCR was performed using SYBR Green qRT-PCR kit (takara, Japan) and ABI step one plus real-time PCR system (applied biosystems, USA). Each sample was in triplicate with β-actin as a reference. The sequences of primers used were given in Table S1.

2.6. Hydrogel regulation of the decomposition/anabolic balance of nucleus pulposus cells

1×10^5 to 5×10^5 cells were seeded into 6-well plates, containing enough culture medium to achieve a cell density of 30%-50%. Agomir874 loaded hydrogel and simple Agomir-874 solution was added to different wells and incubated for 48 h at 37°C. The concentration of Agomir874 was controlled at 200nM.

2.7. Western blot

Proteins were extracted and quantified using a BCA Protein Quantitation Kit. Then the protein samples underwent SDS gel electrophoresis and the target proteins were transferred to PVDF film (0.45 μm, Millipore). The film was first blocked using skimmed milk powder, then incubated overnight using the target antibody (MMP1, MMP2, MMP13, COL II, Sox-9) at 4°C. The film was washed using Tris-buffered saline tween (TBST) and then incubated with species-specific secondary antibodies labeled with horseradish peroxidase (HRP). Enhanced chemiluminescence detection system was used to observe the antigen-antibody complex. The signal intensity was quantified using ImageJ.

3. In vitro single-segment intervertebral biomechanical studies

Single-segment intervertebral samples were separated from SD rats. Briefly, SD rats were anesthetized and the tails were removed for sample preparation. After removing the skin, the tails were washed for three times using 1× PBS solution. The fifth caudal vertebra and sixth caudal vertebra were clipped through center using a rongeur and C5-6 intervertebral disc was ready for experiments. The samples were divided into 5 groups: control, Agomir874@PEG, PEG, Agomir, acupuncture, and each group contained 3 samples each. Each group was punctured using 18G needle apart from the control group. Needles were punctured into the center of nucleus pulposus and stayed for 30 s before removal. Then, 0.05 ml of the material (Agomir874@PEG, PEG, Agomir, and PBS) were injected into intervertebral disc using a 1 ml of injector. Samples were then tested using a Universal testing machine (HY-1080, Hengyi, Shanghai, China) (Figure 4a).

4. In vivo evaluation

4.1. Animal studies

All animal experiments were approved by the Animal Ethics Committee, Ruijin Hospital, Shanghai. 8 weeks SD rats were purchased from Shanghai JSJ lab. Each cage contained three rats and the rats intaken food and water freely all the time. The room temperature remained at 22±2 ℃. After two weeks of accommodation, 15 rats weighed 300–350 g were selected and the rat caudal intervertebral degeneration

models were established, using a method previously described. After anesthesia, 18G needles were punctured into C5-6, C6-7, C7-8 and C8-9 intervertebral disc under X-ray. The puncture depth reached centers of intervertebral discs and needles stayed for 30 s before removal (Figure 5a). Then, 0.05 ml of the material (Agomir874@PEG, PEG, Agomir, and PBS) were injected into the intervertebral disc using a 1 ml injector. The concentration of Agomir874 was controlled at 200 nM. All experiments were performed under sterile conditions.

4.2. Radiology evaluation

The X-ray rat experiments were recorded at different time points (0 week, 1 week, 4 weeks, and 8 weeks after surgery). Disc heights were calculated using lateral film. MRI scans were performed on experimental rats instantly after surgery and 8 weeks after surgery. Water content of intervertebral discs were calculated using internal gray scale of intervertebral disc in lumbar sagittal T2W1 MRI. The parameters of MRI were set according to preliminary experiments. Slew rate: 150 mT/m/ms; Gradient field intensity: 30 mT/m. Parameter for spin-echo sequence T2W1: TR, TE = 3500 ms/120 ms, scan matrix: 256×256, reconstruction matrix: 512×512, FOV (mm) =100.00, RFOV (%) = 100.00, slice thickness = 3mm, scan resolution = 0.3mm.

4.3. Histological evaluation

Samples were collected 4 and 8 weeks after surgery under sterile conditiona. Samples were fixed with 4% formamint at 4 ℃ for 48 h and then EDTA decalcification for 4

weeks. After decalcification, samples were rinse overnight with tap water, dehydrated using graded ethanol, vitrification by dimethylbenzene and embedded in paraffin before frozen under −20℃ for 12 h. The samples were cut into 5-μm slices using a Leica histo-cryotome. Slices were baked overnight in thermostat at 60 ℃. H&E staining were used to compare the changes in intervertebral disc structure. Saffron O staining was used to observe collagen remodeling and composition. Immunohistochemistry was used to quantify the expression of Col II and aggrecan.

5. Statistical analysis

All data were expressed as mean value±standard deviation (SD). SPSS 19 software package (SPSS, IBM, USA) was used for statistical analysis. On-way analysis of variance (ANOVA) and Student's t-test was used to measure the statistical significance of the difference. $P<0.05$ was considered statistically significant while $P>0.05$ was considered none significant (ns) (*$P<0.05$, **$P<0.01$, ***$P<0.001$, and ****$P<0.0001$).

SUPPLEMENTARY FIGURES

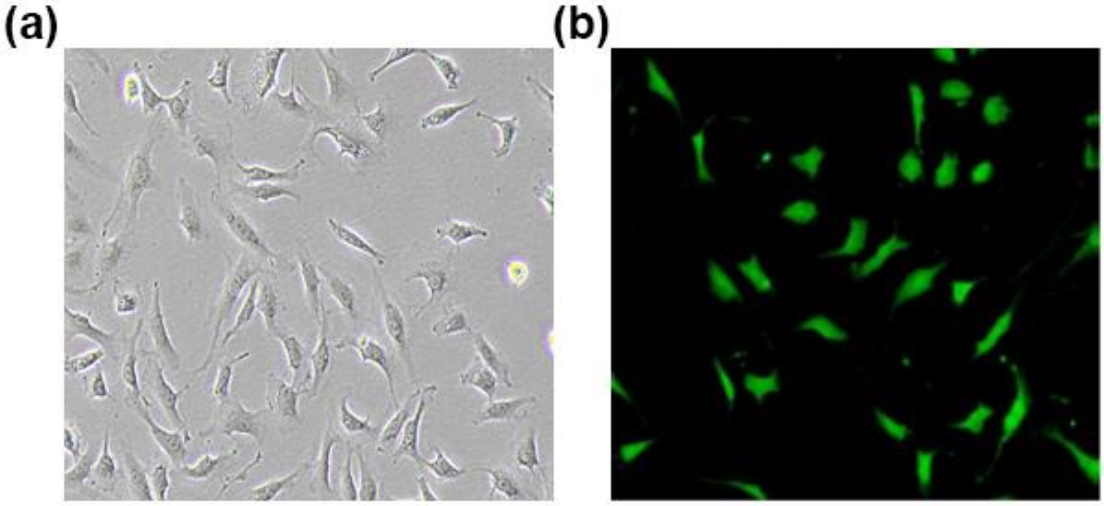

Figure S1. The morphological images of primary nucleus pulposus cells. (a) Morphology image under light microscopy of NPCs. (b) SOX-9 immunofluorescence staining image of NPCs.

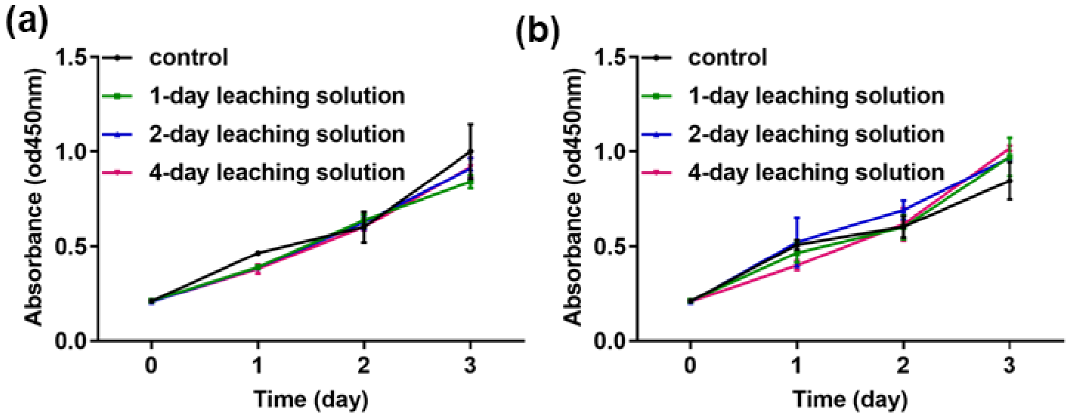

Figure S2. Cell viability in different leaching solution of PEG-Ag hydrogel detected by CCK-8 assay, cell viability was tested every day for 3 days in a row. (a) Cell viability of NPCs of rats. (b) Cell viability of MC3T3-E1 of mouse.

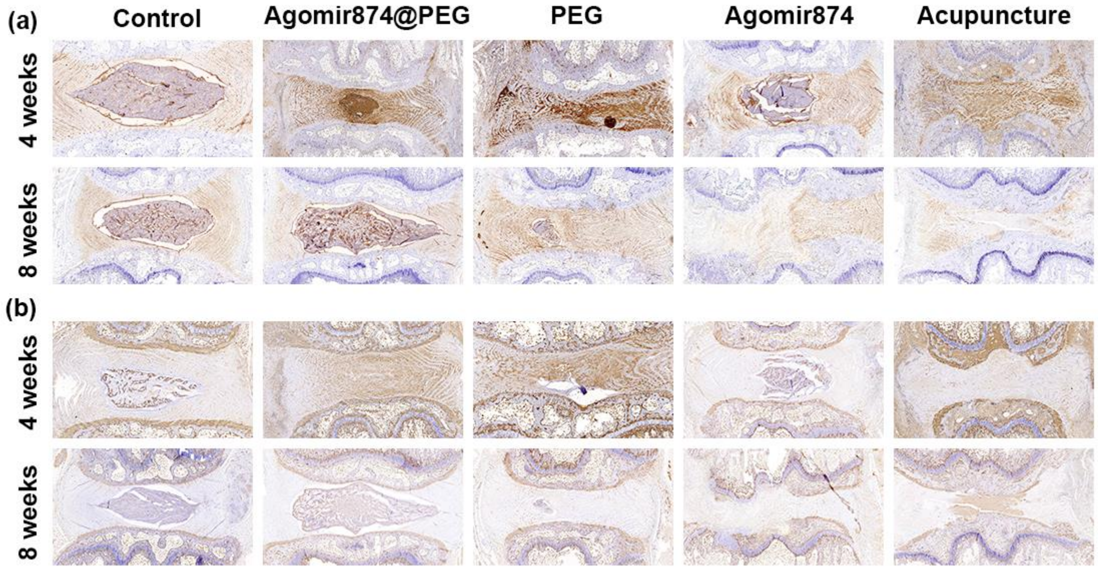

Figure S3. Immunohistochemistry images of animal experiments. (a) The immunohistochemistry of type II collagen images of different groups at different time points. (b) The immunohistochemistry of aggrecan images of different groups at different time points.

Table S1. Primers used in real-time PCR.

Gene	Primer	Sequence
β-actin	FORWARD	TGTCACCAACTGGGACGATA
	REVERSE	GGGGTGTTGAAGGTCTCAAA
mmp1	FORWARD	TGTTCGCCTTCTACAGAGGAGACC
	REVERSE	TGTCGGTCCACGTCTCATCCAG
mmp2	FORWARD	AGCTGTGGACTCTAGGAGAAGGAC
	REVERSE	GAACACCAGAGGAAGCCGTCAC
mmp13	FORWARD	AACCAAGATGTGGAGTGCCTGATG
	REVERSE	CACATCAGACCAGACCTTGAAGGC
col2	FORWARD	ACGCTCAAGTCGCTGAACAACC
	REVERSE	ATCCAGTAGTCTCCGCTCTTCCAC
sox-9	FORWARD	TCAACGGCTCCAGCAAGAACAAG
	REVERSE	CTCCGCCTCCTCCACGAAGG
